# Correction to: Health outcomes in COPD smokers using heated tobacco products: a 3-year follow-up

**DOI:** 10.1007/s11739-022-03043-4

**Published:** 2022-06-30

**Authors:** Riccardo Polosa, Jaymin B. Morjaria, Umberto Prosperini, Barbara Busà, Alfio Pennisi, Gualberto Gussoni, Sonja Rust, Marilena Maglia, Pasquale Caponnetto

**Affiliations:** 1 Department of Clinical and Experimental Medicine, University of Catania, Catania, Italy; 2 Centre for the Prevention and Treatment of Tobacco Addiction (CPCT), Teaching Hospital “Policlinico-V. Emanuele”, University of Catania, Catania, Italy; 3 Center of Excellence for the Acceleration of Harm Reduction (CoEHAR), Università di Catania, Catania, Italy; 4 Department of Respiratory Medicine, Royal Brompton and Harefield Hospital Foundation Trust, Harefield Hospital, Harefield, UK; 5 Hospital “San Vincenzo”, Taormina, Italy; 6 UOC Farmacia Ospedaliera, Hospital ARNAS Garibaldi, Catania, Italy; 7 Department of Respiratory Medicine, Hospital Clinics “Musumeci-Gecas”, Catania, Italy; 8 Department for Clinical Research “Centro Studi” FADOI (Scientific Society of Internal Medicine), Milan, Italy; 9 UOC Medicina Interna e Urgenza, AOU “Policlinico-V. Emanuele-San Marco”, Via S. Sofia, 78-Ed. 4, p. 2, Stanza 78, 95100 Catania, Italy

## Correction to: Internal and Emergency Medicine 10.1007/s11739-021-02674-3

In the acknowledgment section for JBM in the original publication of the article, the donation was not made to ASH but various charities. Hence, the correct sentence should read as “All proceeds of the work were donated to a number of charities”.

The original article has been corrected.

